# Spores of *Beauveria bassiana* and *Trichoderma lignorum* as a bioinsecticide for the control of *Atta cephalotes*

**DOI:** 10.1186/s40659-019-0259-y

**Published:** 2019-09-17

**Authors:** Fabian Felipe Fernandez Daza, Ginna Rodriguez Roman, Marino Valencia Rodriguez, Ivan Andres Gonzalez Vargas, Heiber Cardenas Heano, Marney Pascoli Cereda, Raul Alberto Cuervo Mulet

**Affiliations:** 1 Biotechnology Group, Universidad de San Buenaventura-Cali, Av 122# 6-65, Cali, 76001 Colombia; 2 Economy, Management, Land and Sustainable Development Group, Universidad de San Buenaventura-Cali, Av 122# 6-65, Cali, 76001 Colombia; 3 Microbiology Industry and Environment Research Group (GIMIA), Universidad Santiago de Cali, Av 5 #62-00, Cali, 760035 Colombia; 4 Universidad del Valle, Av 13 #100-00, AA 25360 Cali, Colombia; 5 Valorización del potencial de leveduras aisladas de la Region Centro-Oeste para uso industrial, Universidad Catolica Dom Bosco-Brasil, Campo Grande, Brazil

**Keywords:** Entomopathogenic fungi, Biological control, Bioinsecticide, Biological risk, Field validation

## Abstract

**Background:**

The leafcutter ant (*Atta cephalotes*) is associated with losses in the agricultural sector, due to its defoliating activity; for its control, biological, mechanical and chemical methods have been developed, the latter associated with adverse effects on human and environmental health. This research validated in the field for the control of the leafcutter ant (*A. cephalotes*) using a mixture of *Beauveria bassiana* and *Trichoderma lignorum* spores.

**Methods:**

The effectiveness from the combination of spores of *B. bassiana* and *T. lignorum* with an initial concentration of 2 × 10^9^ spores/ml, in the following proportions of *B.* bassiana and *T. lignorum*, A (1:1), of each fungus. It was evaluated within the university campus, comparing it with two commercial formulations, Mycotrol (*B. bassiana*) and Mycobac (*T. lignorum*). Additionally, this formulation was evaluated in 49 nests distributed 16 in 14 locations in Colombia. The formulation application was carried out by direct application, using a pump at a speed of 10 ml/m^2^. The effectiveness was estimated from the reduction of the flow of ants, evaluating the statistically significant differences using the ANOVA and Tukey-test.

**Results:**

Effective control of 90% of the nests was observed in the field phase in 60 days, except in nests with areas > 50 m^2^ that were located in regions with high rainfall (annual average precipitation above 7000 mm), such as Buenaventura.

**Conclusions:**

In this work, it was demonstrated that the combination of *B. bassiana* and *T. lignorum* spores represent a viable alternative for the control of the leafcutter ant, in which the effectiveness is related to several factors, including the size of the nest and the rainfall in the area.

## Background

The leafcutter ant *Atta cephalotes* (Linnaeus, 1758) is distributed throughout Colombia, especially in the Pacific and Andean regions. Due to their defoliating activity, *A. cephalotes* affects crops such as cotton (*Gossypium* L.), cocoa (*Theobroma cacao* L.), yucca (*Manihot esculenta* Crantz), corn (*Zea mays* L.), rubber (*Hevea brasiliensis* Mull. Arg.), sugar cane (*Saccharum officinarum* L.) and citrus fruits [1, 2] and is considered a pest species. It is estimated that *A. cephalotes* can consume between 50 and 150 kg of plant material per day. In addition, damage to buildings and roads has been reported due to the destabilization of soils from the removal of land during the construction of nests [3].

To control leafcutter ants, mechanical, chemical and biological methods have been developed. Mechanical control consist of the removal of the nests with the extraction of the queen ant and the destruction of the breeding chambers; this method has an effectiveness of approximately 30% [3]. Chemical control is based on the application of insecticides, which generally have the active components sulfluramid, fipronil, deltamethrin, chlorpyrifos and fenitrothion. These insecticides may present risks to human health [4, 5], may cause problems associated with bio-concentration, bio-accumulation and bio-magnification in ecosystems [6–8], and may be associated with the development of resistance in economic impact pests such as ticks [*Rhipicephalus* (*Boophilus*) *microplus*] [5], mites (*Phenacoccus solenopsis*) [9] and fire ants (*Solenopsis invicta*) [10]. In some cases, these pesticides are applied by thermal fogging, which increases soil contamination and does not affect all of the ants in the nest. Biological methods include the application of plant extracts [11, 12] and the use of trapping crops [13], which temporarily reduce the population of the nest without definitively eliminating it; thus, the accompaniment of other control mechanisms to improve effectiveness is recommended [3].

For all of the above reasons, the use of biological controls in agriculture has grown continuously, especially biocontrols composed of the spores of filamentous fungi such as *Beauveria bassiana*, an entomopathogenic fungus, which infects the insect by adhesion to the cuticle insect host by adhesion proteins (Mad 1, Mad 2—*Adhesin*-*like proteins*; Hyd 1, Hyd 2, Hyd 3—*Hydrophobins* [14, 15], followed of the formation of the appressorium, which generates the breakdown of chitin by means of a combination of mechanical strength and enzymatic degradation (proteases, chitinases and lipases) [16], which also focuses enzymatic activity through the production of a mucilage on the site of contact, isolating the point from the outside [17]. The evasion of the immune system is mediated by the expression of Mcl1 proteins [18], the secretion of destruxins A and E, production of trehalase and expression of faith genes involved in the cycle cell, allowing rapid multiplication and differentiation of hyphae in the host’s hemolymph, leading to host mummification [14, 19–21]. On the other hand, the Trichoderma genus is characterized by its Mycoparasite activity, which is initiated by the chemotropic growth towards the host that is recognized through lectin-carbohydrate interactions [22–25]. The adhesion of the hyphae is done by the formation of appressoria, wrapping around the hypha of the infected fungus to which they adhere to the hyphae, and where it usually covers and penetrates the hyphae of the parasitized fungus, followed by degradation of the cell walls, which occurs due to the production of extracellular lytic enzymes (chitinases, glucanase, xylanase, *N*-acetylglucosaminidase, and proteases), which is regulated by the MAPK pathway [26]; These enzymes degrade the cell walls of the host and allow the penetration of the antagonist hyphae, thus forming pores allowing access to the cytoplasm, from which it will extract the nutrients required for its development [27].

Due to the negative impact of leafcutter ants on food security as well as the impact on local economies, we evaluated a bioinsecticide composed of the spores of *B. Bassiana* (ATCC MYA-4886) and *Trichoderma lignorum* (ATCC 8751) for the control of the leafcutter ant (*Atta cephalotes)* under field conditions.

## Methodology

In this study, strains of *B. bassiana* (ATCC MYA-4886) and *T. lignorum* (ATCC 8751) were grown in PDA medium (agar, potato, dextrose) and were incubated at 28 ± 1 °C for 5 days. The spores were collected by scraping the surface and deposited into the YPD medium (yeast extract, peptone, dextrose). A concentration 1 × 10^6^ spores/ml is confirmed using a Neubauer hemocytometer [28], to prepared the inoculum (500 ml). The inoculum was placed in the fermenter (BIOSTAT^®^ B), Adding YPD, up to 4 l and grown by 4 days at 20 rpm of agitation, 30 °C and pH 5.4 [29, 30]. Every 24 h sample of 5 ml was taken for the spore count and grown in PDA medium used for the identified accord to the key described by Barnett and Hunter [31]. Finally, it is centrifuged at 8000 rpm for 15 min, discarding the supernatant, washing the spores and storing them in sterile distilled water.

The formulation used was the combination of spores of *B. bassiana* and *T. lignorum* with an initial concentration of 2 × 10^9^ spores/ml which include Twen20, Sorbitol, Sodium saccharin, Sodium benzoate, Citric acid, Methylparaben and sodium cyclamate. Viability, purity, pathogenicity, and pH of the formulation are described in Fernandez et al. [32].

### Tests: University campus

This phase was carried out in 11 nests (Table 1) present on the campus of the University of San Buenaventura-Cali. We inoculated 10 ml/m^2^ of the following solutions at weeks 2, 7 and 11: Mycotrol (*B. bassiana*), Mycobac (*T. lignorum*), and the formulation with the greatest pathogenicity at a final concentration of 1 × 10^9^ conidia/ml. For each nest, the flow of ants was estimated [33–35], measured as the number of ants that emerged over 5 min intervals (from 5:00 to 6:00 p.m.). Each count was performed in triplicate, daily for 14 weeks. Nest areas were also estimated.

**Table 1:** Characteristics of the nests used for the Tests on university campus

Characteristics	Mycotrol			Mycobac			Control		Formulation A		
	R1	R2	R3	R4	R5	R6	R7	R8	R9	R10	R11
Length	7.23	6.45	8.33	4.57	5.6	6.2	7.25	6.23	6.32	4.15	5.43
Width	3.15	2.78	4.12	3.37	3.17	3.30	2.8	4.27	2.75	4.76	3.86
Area (m^2^)	22.77	17.93	34.32	15.40	17.75	20.56	20.3	26.60	17.38	19.754	20.96

*R* nest evaluated

### Field phase

This phase was carried out to observe the reproducibility of the university campus test, in different regions of Colombia. The external field tests were performed in 49 nests in 14 Colombian localities with different soils, climates (temperature) and rainfall (precipitation) (Table 2). The experimental bioinsecticide (formulation A) was applied as previously described. The effectiveness was estimated as the average flow of ants (percentage), calculated as the ratio of initial percentage to the final observed percentage for each period (week 4, 8 and 12). Additionally, the intervened nests were classified according to area (small, < 10 m^2^; medium, 10 m^2^ to < 50 m^2^; and large, > 50 m^2^), and 14 nests (to which the bioinsecticide was not applied) were included as a negative control. At the end of the monitoring phase (12 weeks), 14 dead individuals were collected, which were deposited into Petri dishes containing PDA and YPDA and were incubated for 10 days at 30 °C. Plates were monitored for fungal growth.

**Table 2:** Geographical distribution of intervened nests in field test

Department	Location	Elevation (masl)	Annual rainfall	R. H	T (°C)	Coordinates of intervened nests
Choco	Lloró	65	7774	84	26.9	5°29′46.0″N 76°32′06.5″W 5°29′59.0″N 76°32′19.7″W 5°30′53.3″N 76°32′32.9″W 5°31′11.7″N 76°32′36.1″W
	Atrato	43	12,000	84	30	5°31′30.5″N 76°38′08.6″W 5°31′54.1″N 76°38′29.4″W 5°31′47.9″N 76°38′34.9″W
Valle del Cauca	Buenaventura	7	7328	60	30	3°57′14.8″N 76°58′45.4″W 4°00′00.3″N 76°57′33.7″W 3°59′50.1″N 76°58′45.4″W
	Bugalagrande	941	941	40	28	4°11′27.7″N 76°10′07.6″W 4°12′59.5″N 76°09′15.5″W 4°11′42.1″N 76°09′50.9″W
	Caicedonia	1100	1872	60	22.4	4°19′48.7″N 75°48′42.7″W 4°20′06.6″N 75°48′48.4″W 4°19′27.7″N 75°48′58.4″W
Antioquia	Yolombo	1450	2837	57	21	6°36′49.7″N 74°59′46.5″W 6°35′07.5″N 74°58′30.2″W 6°35′24.4″N 74°58′12.1″W
	Girardota	1425	1821	89	22.2	6°23′06.4″N 75°27′47.8″W 6°23′20.3″N 75°26′55.8″W 6°22′30.5″N 75°27′00.6″W 6°22′44.8″N 75°26′45.6″W
Huila	Tesalia	830	1495	71	23.7	2°29′10.1″N 75°43′02.0″W 2°29′08.7″N 75°43′03.6″W 2°28′33.7″N 75°44′23.3″W 2°28′33.3″N 75°44′23.7″W
	La Argentina	1560	1813	NA	18	2°11′39.1″N 75°59′18.1″W 2°12′15.2″N 75°58′35.3″W 2°12′42.2″N 75°58′35.0″W
	La plata	1050	1499	68	22.7	2°27′34.1″N 75°50′08.0″W 2°22′08.3″N 75°54′28.3″W 2°22′09.6″N 75°54′32.3″W
Cundinamarca	Tocaima	400	1260	NA	27	4°27′12.2″N 74°37′11.0″W 4°27′05.5″N 74°38′23.7″W 4°25′47.0″N 74°38′49.8″W 4°26′19.9″N 74°37′55.5″W
Sucre	Sincelejo	213	1164	54	26.6	9°18′24.4″N 75°21′29.1″W 9°18′01.0″N 75°21′35.8″W
Magdalena	Santa Marta	2	512	52	28.3	11°17′33.7″N 4°00′01.5″W 11°17′37.5″N 4°00′00.8″W 11°17′40.8″N 4°00′00.9″W
Córdoba	Montería	18	1225	66	27.4	8°46′41.4″N 75°58′26.3″W 8°47′59.3″N 75°57′17.1″W 8°48′34.6″N 75°56′20.6″W

*R. H* relative humidity, *T* temperature in °C, *masl* meters above sea level

### Analysis of results

In tests: University campus and field phase, each nest subjected to the same treatment was considered a replicate, and each subjected ant flow (performed in triplicate each day, for 14 weeks) was considered a repetition. The treatment efficacy was estimated as the percentage value of the ratio between post-exposure and pre-exposure ant flow. The differences between the treatments were evaluated by analysis of variance (ANOVA) and Tukey’s test (α = 0.05) (SPSS 20.0.) using the average daily ant flow counts, obtained for each week in the field tests, with previous compliance of the assumptions of variance homogeneity and normal distribution.

### Ethical considerations

For the execution of this investigation, the ethical endorsement Ing7I15 was granted by the research ethics committee of the university of San Buenaventura-Cali. Animal ethics endorsement was granted by the CIECUAE committee of the university ICESI no. 0023 of 2017.

## Results

### Tests: University campus

A total of 11 nests were tested, in which 50% had three exits and entrances. The nests had an average length of 5.86 m, an average width of 3.38 m, and mound areas between 15 and 23 m^2^. The temperature was maintained in a range between 22 and 32 °C. We observed a flow of ants between 90 and 103, which were average values for week 1 (without inoculation). The greatest reduction in the flow of ants compared to the initial flow was observed during weeks 6 (29.54% to 52.26), 12 (66% to 97%) and 14 (87.9 to 100%), and an increase of 30.6 ants was observed in the controls. The greatest effectiveness corresponded to formulation A (spores of *B. bassiana* and *T. lignorum*, patent *A01N 65/00*), showing statistically significant differences between the control and the different treatments commercial bioformulations Mycontrol (*B. bassiana*) and Mycobac (*T. lignorum*), evaluated in the local field test (Table 3).

**Table 3:** Effectiveness of the formulations in the Tests on university campus

Treatment	n	Week 1	Week 6^1^	Week 12^2^	Week 14^3^
*Trichoderma lignorum* (*Mycobac*)					
R4	5	100	66.67 + 2.09 bc	32.07 ± 1.52 de	22.83 + 0.76 d
R5	5	100	71.34 + 3.29 c	31.96 ± 2.39 d	23.60 + 0.8 d
R6	5	100	78.91 +2.83 d	35.87 ± 1.88 e	25.87 + 0.91 c
*Beauveria Bassiana* (*Mycontrol*)					
R1	5	100	59.50 + 4.62 b	13.45 +2.02 b	13.90 + 0.90 b
R2	5	100	48.82 + 4.6 a	15.65 +2.45 b	12.90 +1.58 b
R3	5	100	51.61 + 3.79 a	16.27 + 1.65 b	10.04 + 0.71 f
Formulation A					
R9	5	100	48.6 + 1.96 a	3.88 + 0.86 a	0
R10	5	100	50.84 + 150 a	1.86 + 1.73 a	0
R11	5	100	45.22 + 0.86 a	2.72 + 1.57 a	0
Control					
R7	5	100	101.57 + 4.10 e	122.85 + 1.34 c	130.06 + 1.65 e
R8	5	100	109.02 + 4.34 e	124.27 +1.83 c	131.31 + 0.82 e

The values correspond to the flow rate (percentage) of ants, calculated with respect to the value of week 1, ± standard deviation, *R* nest

Equal letters identify groups with similar means (Tukey α = 0.05)

^1^F = 3076.408 df = 10 p = 0.0001

^2^F = 3080.971 df = 10 p = 0.0001

^3^F = 11,934.834 df = 7 p = 0.0001

### Field phase

We observed differences in effectiveness associated with nest size in regions where eradication of nests > 10 m^2^ was achieved in an average of 4 weeks (30 days), eradication of medium-sized nests was achieved in 8 weeks (60 days), and a reduction of at least 20% of ants in large nests was recorded after 8 weeks, which exhibited no ant flow after 12 weeks. The variations among the groupings between nest sizes was associated with differences between them in each locality (Table 4), in contrast to the nests from the localities of Buenaventura, Lloró and Atrato, where the bioinsecticide was not effective.

**Table 4:** Effectiveness of the formulation A in the field, by location in the different weeks of evaluation

Location	n	Small nest (Área < 10 m^2^)			Medium nest (10 m^2^ < 50 m^2^)			Large nest (Área > 50 m^2^)		
		Week 4	Week 8	Week 12	Week 4	Week 8	Week 12	Week 4	Week 8	Week 12
Antioquia yolombo	6	2.3 ± 1.00	0	0	15.4 ± 1.92	0.1 ± 0.12	0	60.8 ± 1.07	20.4 ± 0.77	0
Antioquia Girardota	3	0	0	0	13.2 ± 0.68	0	0	56.3 ± 0.80	12.8 ± 0.72	0
Choco Atrato	3				68.2 ± 0.88	100	100	96 ± 1.11	100	100
Choco Lloro	4				48.1 ± 0.99	100	100	93.8 ± 1.68	100	100
Córdoba Montería	3	0	0	0						
Cundinamarca Tocaima	4	1.6 ± 1.31	0	0	8.2 ± 1.10	0.6 ± 1.10	0			
Huila La Argentina	4	0	0	0	14.9 ± 2.31	0	0			
Huila La Plata	3	0	0	0	29.2 ± 0.52	7 ± 3.49	0			
Huila Tesalia	4				23 ± 3.72	5 ± 2.03	0	63 ± 1.28	19 ± 3.24	0
Magdalena Santa marta	3	0.2 ± 0.64	0	0	13 ± 1.31					
Sucre Sincelejo	3	0	0	0						
Valle Caicedonia	3	0	0	0	27 ± 2.34	4.2 ± 0.50	0			
Valle Bugalagrande	3	0	0	0	0.6 ± 1.77	0	0			
Valle Buenaventura	3				60 ± 2.08	100	100	94	100	100

The values correspond to the flow rate (percentage) of ants, calculated with respect to the value of week 1, ± standard deviation

## Discussion

In the present study, it was demonstrated that treatment with a formulation containing conidia of *B. bassiana* and *T. lignorum* presents insecticidal activity against *A. cephalotes* and can be considered for the control of this agricultural pest. Control the leafcutter ant (*A. cephalotes*) can be achieved by controlling/eliminating the food source (*Leucoagaricus gongylophorus* [36]), or the queen; unique for each anthill, responsible for generating the individuals [11] of the different castes required for the operation of the anthill [37]. The tests: University campus showed that formulation A, achieved a reduction in the flow of ants (100%) in the anthills evaluated at week 14, contrasting with that obtained by the commercial biological formulations Mycobac (*T. lignorum*) and Mycotrol (*B. bassiana*), where reduction in the flow of ants was observed between 74 and 86% respectively; These differences could be associated with the mechanism of action of the fungal species. In the case of Mycobac, the controlling effect on the antigenic population is determined by the capacity of *T. lignorum* on *L. gongylophorus* [38–41]; the progressive infection would explain the reduction in observed ant flow (between 74.13 and 77.17%) in the trials with this bioform. Additionally, in Mycotrol, the reduction of ant flow is related to the entomopathogenic nature of *B. bassiana* [41, 42], which can infect the ant population or the queen ant. It was observed in the test: university campus, the reduction of the flow of ants between 86.10 and 89.96%, without identifying the elimination of the anthill, which suggests that there was no infection in the queen ant. The literature reports other studies where they identified different factors that inhibit the controlling activity of *B. bassiana*, among which are the presence in the cuticle of insects, presence of bacteria such as *Wolbachia*, *Streptomyces*, secretion of antifungal substances [19, 37, 43]. The presence of yeast species that inhibit the growth of entomopathogenic fungi [26, 44] that, although they do not completely inhibit the entomopathogenic fungus, allow the anthill to opt for other strategies to contain the infection, among which are the removal of infected individuals, among others [19]. The differences between the commercial biological formulations of Mycobac (*T. lignorum*) and Mycotrol (*B. bassiana*) with respect to the formulation A, could be associated to that in the formulation A the spores of these two fungal species are mixed (*B. bassiana* and *T. lignorum*), which allows the involvement of the ants and the symbiont *L. gongylophorus* [36], increasing the effect on the control of ant flow.

Different studies have evaluated the activity of fungal spores as inhibitors of leafcutter ants. For example, the effect of spores of *B. bassiana* strain ZGNKY-5 in a liquid suspension (1 × 10^8^ spore/ml) on *Solenopsis invicta* nests was evaluated. These nests were located in Panyu, Guangzhou, and the biocontrol achieved 100% effectiveness at doses between 5 × 10^10^ at 1 × 10^11^ spore/ml (500 to 1000 ml), which were applied to nests in areas smaller than 10 m^2^ [45]. Additionally, a study on *A. cephalotes* nests (areas < 50 m^2^) located in Girardota (also evaluated in this study) and Barbosa, evaluated spores of *M. anisopliae* and *T. viride*, which showed 100% eradication effectiveness, except treatment with *T. viride* in the locality of Girardota. This result was observed after 42 weeks of evaluation, with effects observed after week 12. The evaluation times were longer than those in our tests, in which similar effects were achieved, even in nests greater than 50 m^2^ in 12 weeks of evaluation. The reduction in time may be associated with the repeated inoculation of the bioinsecticide (weeks 1, 4 and 8), which increases the dose and presence of spores in the symbiotic fungi chambers. Here, the workers increase removal activity, which drastically reduces the flow of workers on the surface [46].

In this study, we observed that the bioinsecticide was not effective in the nests located in the localities of Buenaventura, Lloró and Atrato. These localities are in the Colombian Pacific region and are characterized by rainfall in annual average, of more than 7000 mm^3^. It rained during the application of the bioinsecticide, and as a result, the surface and entrance of the nests were washed out, which decreased the quantity of spores. Additionally, excess rainfall reduced the dosage because in nests with areas greater than 50 m^2^, the spores were effectively removed from the chambers by worker ants [46]. The effect of rain and the reduced effectiveness of the formulations, both experimentally tested in this study, as well as in the chemical control, were shown by the intervened communities.

Finally, reports in the literature indicate that the microbiota present on the surface of the insect, as well as that found at the intestinal level, can behave as a barrier against pathogens, behaving like an extension of the “innate immunity”. Additionally, where the in vitro pathogenesis of an entomopathogenic fungus species is evaluated, they require the removal of the accompanying microbiota [35, 47], becoming a bias regarding the pathogenicity assessment of the entomopathogenic fungus; this is how the accompanying microbiota in the insect to be controlled could be part of the explanation of what was observed in field tests, where the control values (measured at exposure time) are usually higher than those found in the laboratory [43].

## Conclusions

In the present study, it was demonstrated that treatment with a formulation (A) containing conidia of *B. bassiana* and *T. lignorum* presents insecticidal activity against *A. cephalotes* and can be considered for the control of this agricultural pest, presenting greater effectiveness than commercial biocontrollers of Mycobac (*T. lignorum*) and Mycotrol (*B. bassiana*). We showed the effectiveness of the bioinsecticide is related to the size of the nests; is necessary to carry out serial dosages of the experimental insecticide to guarantee the control of *A. cephalotes.* It was not effective in nests located in regions characterized by an annual rainfall of more than 7000 mm^3^. It should be noted that the experimental formulation made with the spores of the filamentous fungi of *B. bassiana* and *T. lignorum* was patented (A01N 65/00) and the transfer of technology is expected to all farmers that are affected by this pest.

## Data Availability

Not applicable.

## Abbreviations

PDA: medium agar, potato, dextrose
YPD: yeast extract, peptone, dextrose
AN: nutritive agar
CIECUAE: Comité Institucional de Ética para el Cuidado y Uso de Animales en Experimentación
